# NK Cell Dysfunction and Checkpoint Immunotherapy

**DOI:** 10.3389/fimmu.2019.01999

**Published:** 2019-08-21

**Authors:** Jiacheng Bi, Zhigang Tian

**Affiliations:** ^1^Shenzhen Laboratory of Antibody Engineering, Institute of Biomedicine and Biotechnology, Shenzhen Institutes of Advanced Technology, Chinese Academy of Sciences, Shenzhen, China; ^2^Division of Molecular Medicine, Hefei National Laboratory for Physical Sciences at Microscale, The CAS Key Laboratory of Innate Immunity and Chronic Disease, School of Life Sciences, University of Science and Technology of China, Hefei, China; ^3^Institute of Immunology, University of Science and Technology of China, Hefei, China

**Keywords:** inhibitory receptors, checkpoint blockade, immune evasion, immune tolerance, regional immunity

## Abstract

NK cells play important roles in the innate immune responses against tumors. The effector function of NK cells relies on the integration of activating and inhibitory signals. Emerging checkpoint receptors and molecules are being revealed to mediate NK cell dysfunction in the tumor microenvironment. Inhibition of some NK cell surface checkpoint receptors has displayed the potential to reverse NK cell dysfunction in tumors, and to boost anti-tumor immunity, both in clinical trials (anti-KIR and anti-NKG2A), and in preclinical studies (e.g., anti-TIGIT, and anti-CD96). To fully exploit the potential of NK–based checkpoint immunotherapy, more understanding of the regional features of NK cells in the tumor microenvironment is required. This will provide valuable information regarding the dynamic nature of NK cell immune response against tumors, as well as novel checkpoints or pathways to be targeted. In this Review, we discuss recent advances in the understanding of NK cell dysfunction in tumors, as well as emerging strategies of NK-based checkpoint immunotherapy for tumors.

## Introduction

Limited response rates of T cell–based checkpoint immunotherapies against CTLA-4 (CytoToxic Lymphocyte Antigen 4) and/or PD-1 (Programmed-cell Death protein 1)/PD-L1 indicate that additional checkpoints/pathways exist to suppress efficient tumor immunity (1–3). Moreover, tumors usually escape T cell immune surveillance by downregulating the expression of major histocompatibility complex (MHC) class I to compromise the tumor antigen presentation pathway (4–6), making these tumors difficult to be recognized by T cells. However, these tumors are highly susceptible to NK cell elimination via the “missing-self” recognition (7, 8). On the other hand, tumors with low mutational loads usually trigger less effective T cell responses than tumors with high mutational loads (9–11). Nevertheless, even tumors with low mutational loads should be recognized and killed by NK cells. These alternative features suggest that NK cells could serve as the major anti-tumor effector cells where tumors should develop mechanisms of escaping T cell surveillance, thus providing additional benefits to T cell–based immunotherapies. Therefore, NK cells represent an emerging target for tumor immunotherapies (12, 13).

On the other hand, multiple intrinsic, and extrinsic immune suppressive checkpoints/pathways exist to prevent NK cells from fully displaying the anti-tumor potentials in the tumor microenvironment (14, 15). Among these checkpoints/mechanisms that inhibit tumor-associated NK cell functions, targeting some of the checkpoint receptors of NK cells by monoclonal antibodies has been shown to unleash the anti-tumor effector function of tumor-associated NK cells, highlighting NK cells as a potential target for checkpoint immunotherapy. However, a lot of further work lies ahead to unveil the dynamics of anti-tumor NK cell responses, as well as the regional features of tumor-infiltrating NK cells, not only in the tumor immune suppression landscape, but also in the settings of tumor immunotherapies. Unless we have a better understanding of the basic biology of tumor-associated NK cells, we cannot rationally design strategies that efficiently harness the anti-tumor potential of NK cells.

## Role of NK Cells in Tumor Immunity

The roles of NK cells in tumor surveillance are well-established. Since cytolytic activity is one of the features of NK cell effector functions, decreased cytolytic activity of NK cells has been associated with higher tumor incidence (16, 17), indicating that NK cell cytolytic function is normally required for tumor control. Among various types of tumors, the role of NK cells in the control of blood cancers and tumor metastasis is especially well-recognized. For example, the expression levels of NK cell activating receptors, NKp30 and NKG2D, in lymph nodes with metastasis from tumor patients was negatively correlated with the levels of metastasis (18). In patients with metastatic prostate cancers, NK cells from patients with longer overall survival and castration resistance display high expression levels of activating receptors and high cytotoxicity (19). In mouse models of metastasis, the depletion of NK cells, as well as genetic deficiency of IFN-γ or perforin, resulted in higher levels of metastasis in mice (20, 21). Besides the role in the surveillance against blood cancers and tumor metastasis, the infiltration of NK cells into solid tumors also affects the tumorigenesis (22), at least in some tumors from the colon (23), the stomach (24), lung (25), and the renal (26).

In addition to the role of NK cells in direct tumor surveillance, NK cells also contribute to T cell anti-tumor immunity. In mouse models, NK cells facilitated the accumulation of T-bet^+^CD4^+^ T cells in the tumor region (27), promoted the production of effector molecules, TNF-α and IFN-γ, by tumor-infiltrating CD8^+^ T cells, suppressed the expression of exhaustion marker PD-1 on these CD8^+^ T cells (28), and promoted the induction of tumor-specific T cell memory (29). *In vitro* data suggest that NK cells might facilitate the differentiation of anti-tumor Th1 cells via production of IFN-γ in an NKG2D-dependent manner (27). Also, NK cells are required for the accumulation of conventional type I dendritic cells (cDC1) in tumors in mouse models, as NK cells produce CCL5 and XCL1 chemoattractants (30). Such recruitment of cDC1 is critical for T cell anti-tumor immunity. In human cancers, intratumoral CCL5, XCL1, and XCL2 transcripts correlated with gene signatures of both NK cells and cDC1, and were associated with increased overall patient survival (30). This evidence highlights the role of NK cells as a “helper” in formation of an efficient anti-tumor T cell response.

The “helper” effects of NK cells are important in the context of T cell–based checkpoint immunotherapy. Although anti-PD-1 immunotherapy largely targets T cells, the frequency of intratumoral NK cells was found to correlate with patient responsiveness to PD-1 blockade immunotherapy, and with increased overall survival (31). These intratumoral NK cells formed clusters with intratumoral stimulatory dendritic cells, and thus played a role in stimulating anti-tumor T cell activity (31). In line with this, data from mouse models showed that depletion of NK cells abrogated the efficacy of PD-L1 blockade immunotherapy (28). The presence of NK cells prevented formation of a more exhausted status of tumor-infiltrating CD8^+^ T cells even under conditions of PD-L1 blockade, as evidenced by decreased expression of degranulation marker CD107a, and effector cytokines, TNF-α and IFN-γ, and increased expression of exhaustion marker PD-1 by CD8^+^ T cells, after NK cell depletion (28). Therefore, by facilitating an efficient anti-tumor T cell response, NK cells contribute to the PD-1/PD-L1 checkpoint immunotherapy. Also, higher levels of intratumoral NK cells might serve as a biomarker to predict better clinical response to PD-1/PD-L1 checkpoint immunotherapy.

## NK Cell Activation

Unlike T cells that majorly use antigen-specific T cell receptors (TCR) to recognize target cells for activation, the activation of NK cells relies on the integration of signals from an array of cell surface activating and inhibitory receptors (7, 32, 33). NK cell activation receptors (33–36) include CD16, natural killer gene 2D (NKG2D), natural cytotoxicity receptors (NCRs), activating KIRs in humans (Ly49D and Ly49H in mice), CD226, as well as the signaling lymphocytic activation molecule (SLAM) family of receptors (SFRs).

On the other hand, NK cell inhibitory receptors (37–39), potentially druggable targets in tumor immunotherapy, are referred to as “checkpoint” receptors, which involve killer inhibitory receptors (KIRs), CD94/NKG2A, T cell immunoreceptor with Ig, and immunoreceptor tyrosine-based inhibition motif (ITIM) domains (TIGIT), CD96, T cell immunoglobulin- and mucin-domain-containing molecule 3 (TIM-3), PD-1, CTLA-4, lymphocyte activation gene 3 (LAG-3), and V domain immunoglobulin suppressor of T cell activation (VISTA).

The triggering of NK cell activation usually involves two modes: “missing-self” recognition and “induced-self” recognition (8, 40–42). “Missing-self” recognition happens when the target cells display lower or even absent surface expression of MHC I molecules, which is usually linked with viral infection or cellular transformation. This would result in dampened inhibitory signaling from the MHC-I-binding KIRs or CD94/NKG2A (and Ly49 family members in mice), leading to activation of NK cells. Alternatively, “induced-self” recognition requires the engagement of stress-induced or virus-encoded ligands on target cells by germline-encoded activating receptors.

Besides the balance of surface receptors-mediated signaling, priming also affects strength of NK cell effector activity. Stimulation by infections, cytokines [e.g., type I interferon (IFN), interleukin-15 (IL-15), IL-12, IL-18, IL-21 and IL-1β; either alone or in combinations], and pathogen-associated molecular patterns (PAMPs) can prime NK cells by lowering the threshold for further activation (43), and by inducing expression of effector molecules (44, 45).

Downstream of the surface receptors are common signaling molecules that regulate the triggering and strength of NK cell activation and responses upon ligand engagement or cytokine stimulation (13, 46). For NK cell activating surface receptors, downstream signals converge on SH2 domain-containing leukocyte phosphorylation of 76 kDa (SLP-76)-mediated phosphorylation of Vav1, which is negatively regulated by the E3 ubiquitin ligase, Casitas B-lineage lymphoma-b (Cbl-b) (47). On the other hand, IL-15, the NK cell key cytokine signals through the JAK-STAT pathway, which is inhibited by cytokine-inducible Src homology-2 (SH2)-containing protein (CIS) (48). These molecules are also emerging “checkpoints” in tumor immunotherapy.

## NK Cell Dysfunction in Tumors

Upon activation, NK cells normally form conjugations with target cells, and release cytotoxic granules containing perforin and granzymes for target cell lysis (49–51), or induce target cell apoptosis via TNF-α, FasL, and TRAIL (32, 52, 53). Besides, NK cells are responsible for early and rapid production of anti-tumor effector cytokine IFN-γ (54, 55). However, the tumor microenvironment possesses unique regional immune features compared with the peripheral and other immune organs, resulting in the impaired effector functions of tumor-associated NK cells (14, 15). Firstly, NK cells usually display decreased percentages along tumor progression (56). Secondly, the “quality” of single NK cells is also compromised, as shown by lower effector molecules expression of IFN-γ, CD107a, granzyme B, FasL, TRAIL, and perforin in tumor-infiltrating NK cells as assessed by intracellular staining for flow cytometry (27, 57–61). Notably, the decreases in both “quantity” and “quality” of NK cells in tumors were reported to positively connect with each other (62), indicating that the dysfunction of tumor-associated NK cells is multi-aspect.

The suppressed expression of effector molecules by NK cells suggests that the NK-specific signaling/transcriptional program should be altered in the tumor microenvironment. IL-15 is expressed in the tumor microenvironment, required for establishing normal levels of NK cell anti-tumor immune response (63). However, IL-15 signaling is compromised for NK cells in tumors (64). Therapeutic application of exogeneous IL-15 directed to the tumor sites activated and recruited NK cells in mouse models (65), indicating that IL-15 signaling is essential for NK cell anti-tumor immunity. Furthermore, expression of key transcriptional factors, Eomes and T-bet, are also decreased in NK cells in tumor-bearing mice (66, 67). In line with these alterations, tumor-associated NK cells displayed defective maturation status both in mice (64, 68, 69) and in humans (67, 70, 71). Such hypomaturation status of NK cells has been associated with reduced overall survival and relapse-free survival of patients with acute myeloid leukemia (AML) (72). Together, these defects contribute to compromising the effector program of NK cells in tumors.

The effector function of NK cells is sustained by cellular metabolism (73–76). However, in the tumor microenvironment, not only tumor cells (77–79), but also NK cells display dysregulated metabolism (80). The dysregulated metabolic status would lead to the dysfunctional status of NK cells (81–83), as well as other immune effector cells (84). In a KRas -driven tumor model in the lung, fructose-1,6-bisphosphatase (FBP1) was highly up-regulated in lung NK cells from mice bearing advanced lung tumors, compared with lung NK cells from normal mice (80). FBP1 functions as a rate-limiting enzyme in gluconeogenesis, facilitating gluconeogenesis, and inhibiting glycolysis. Up-regulated FBP1 in NK cells in the tumor settings suppressed the glycolysis of NK cells, compromised their viability, and effector functions (80).

NK cell's dysfunctional status in tumors is accompanied by a series of phenotypic alterations. Multiple NK cell activating receptors have been reported to be down-regulated in tumors. For example, NK cells express decreased levels of NKG2D in various types of cancers (57, 59, 85). In addition, DAP10, the adaptor for transducing NKG2D receptor signaling, was also found to decrease in the chronic viral infection setting, and probably also in tumors, which might further add to the compromised NKG2D signaling (86). Other NK cell activating receptors reported to show decreased expression in tumors include CD16 (57), NCRs (57, 59, 85), and CD226 (56, 57, 85, 87). The downregulation of activating receptors could be restored in remission (57), suggesting that this detrimental regulation of NK cell activating receptors expression is an active suppression mechanism by the tumor microenvironment.

The detrimental regulation of NK cell receptors also involves the upregulation of inhibitory receptors. For example, TIGIT expression on mouse NK cells was up-regulated during tumor progression. In humans, the constitutive expression of TIGIT on NK cells was further up-regulated in tumor regions compared with peritumoral regions in colorectal tumors (28). Other inhibitory receptors reported to be upregulated on NK cells in tumors involve CD96 (88), NKG2A (60), and PD-1 (89–92). The immune suppressive cytokines, such as IL-10 and TGF-β, might contribute to the upregulation of inhibitory receptors (60, 93).

One recently discovered aspect regarding the dysfunction of tumor-associated NK cells is the intratumoral differentiation of NK cells. The dissection of the lineage differences between NK cells and ILC1s has been revealed in multiple tissues (94, 95). This complexity has recently been extended to the tumor microenvironment. TGF-β receptor signaling in NK cells was found to mediate the intratumoral differentiation of conventional anti-tumor effector NK cells into ILC1-like cells, which were unable to control tumor growth (96, 97).

## NK–Based Checkpoint Immunotherapy

**Figure 1:**
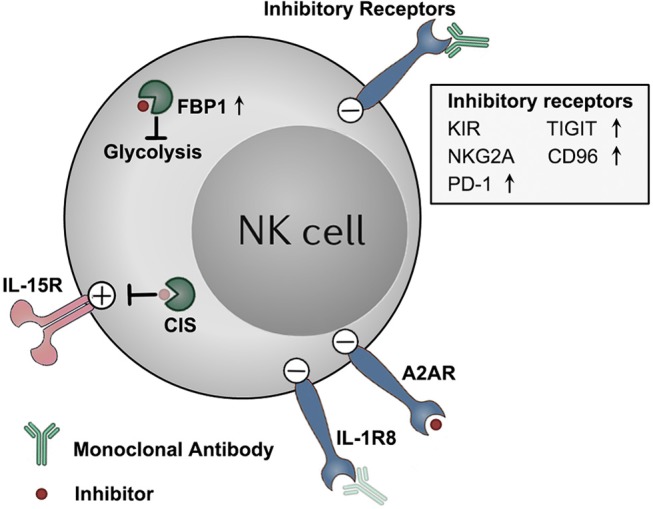
Targeting NK cell dysfunction via checkpoint inhibition tumor immunotherapies. NK cells display a dysfunctional status in tumors, along with detrimental upregulation of some checkpoint molecules (e.g., TIGIT, CD96, PD-1, and FBP1). These molecules, as well as other constitutively expressed checkpoint molecules (e.g., KIRs, NKG2A, A2AR, and IL-1R8), functions to impair the anti-tumor effector potentials of NK cells in tumors. These checkpoint molecules represent potential targets for NK–based immunotherapy. Therapeutically targeting these molecules by blocking monoclonal antibodies or small molecule inhibitors might unleash NK cells from those immune suppressive mechanisms, and boost NK cell anti-tumor activity. Antibody and inhibitor symbols in light color (those for CIS and IL-1R8) indicate that the agents have not been developed yet.

The success of T cell–based checkpoint immunotherapy has revolutionized the treatment for cancer, which has established a concept that unleashing the potential of anti-tumor immunity is capable of controlling tumors. At the same time, the limited responsiveness of current checkpoint immunotherapies is driving the area toward discovering new “checkpoints” on not only T cells, but also on other immune cells, such as on NK cells (Figure 1). On the other hand, recent studies have shed light on the potential of targeting some common signaling regulators to stimulate the anti-tumor activity of immune cells. These molecules have broadened the conventional concept of “checkpoints,” representing new areas in NK–based tumor immunotherapy.

### KIR

The history of NK–based checkpoint immunotherapy started with blocking KIRs on NK cells. Early studies on bone marrow transplantation with acute myeloid leukemia patients showed that 5-year recurrence rate was 75% for donor's NK-KIR matched with recipient's MHC I molecule. However, strikingly, for those mismatched cases, this number dropped to 0% (98). This and other related evidence together indicate that absence of the matching/recognition between NK cell inhibitory receptor KIRs and target cell MHC I molecules would trigger the activity of NK cells, leading to the rejection of these target cells. This lays the rationale of KIR blockade for stimulating NK cell activity against tumors. In clinical trials, KIR blockade showed limited side effects (99). Neither KIR single blockade (NCT01687387), nor combined with anit-CTLA-4 (NCT01750580), displayed better efficacy than anti-CTLA-4 alone. However, combination blockade of KIR and PD-1 showed the trend of increased objective response rate for advanced head and neck tumor patients previously treated with chemotherapy (NCT01714739). Moreover, preliminary data from KIR blockade in combination with 5-azacytidine, a DNA methyltransferase inhibitor, as a therapy for refractory or relapsed AML showed that 20 percent of the first 25 patients responded to therapy, with two patients achieving complete remission (CR) (NCT02399917). These preliminary data have given us confidence on more ongoing clinical trials on KIR blockade alone or combined therapies (e.g., combined with anti-CD20 in NCT02481297, with anti-PD-1, and 5-azacytidine in NCT02599649, or with anti-SLAMF7 in NCT02252263). In addition, more investigations on new indications and the data mining on previous clinical trials might further reveal the potential of KIR blockade in tumor immunotherapy.

### TIGIT

TIGIT is an inhibitory receptor expressed majorly on T cells and NK cells (100, 101). TIGIT binds ligands CD155 and CD112. The inhibitory functions of TIGIT depend on the intracellular ITIM and ITT-like domain (101, 102), on the competition with activating receptors CD226 for ligand interactions (103), as well as on the ligand signaling from CD155 upon interaction (100). The interaction of TIGIT with its ligands suppressed the cytolytic activity and IFN-γ production of NK cells *in vitro* (101, 104). Such effects are critical for its role in maintaining NK cell self-tolerance in acute inflammations (105) and tissue regenerations (106), which, however, in turn lead to the exhausted status of NK cells in tumor settings, promoting the progression of tumors (28).

The expression of checkpoint inhibitory receptor TIGIT on NK cells was up-regulated along tumor progression in mice (28). The tumor-associated expression pattern of TIGIT is not dependent on the adaptive immune system, since TIGIT was also highly expressed on tumor-infiltrating NK cells in SCID mice (28). In humans, TIGIT is constitutively expressed by NK cells, which was up-regulated on NK cells in tumor regions compared with NK cells from peri-tumoral regions (28). Consistently, CD103^+^ tumor resident NK cells displayed higher expression of TIGIT than CD103^−^ circulating NK cells. In addition, tumor-infiltrating NK cells from tumor patients with lymph node invasion expressed higher levels of TIGIT, compared with those from patients without lymph node invasion. These studies demonstrate that the expression of TIGIT on NK cells is associated with tumor progression both in humans and in mice. Importantly, while the expression pattern of two well-established T cell checkpoint receptors, PD-1 and CTLA-4, are more restricted on tumor-infiltrating T cells, TIGIT was highly expressed not only on T cells, but also on NK cells in tumors (28), highlighting TIGIT as a checkpoint receptor more specific to NK cells.

Monoclonal antibody blocking TIGIT reversed the exhaustion status of tumor infiltrating NK cells (28). Such effect adds to the T cell -stimulating effects on both regulatory T cells (107) and effector T cells (103) by TIGIT blockade. With these supportive results from preclinical studies, therapeutic blockade of TIGIT in advanced cancers is currently being tested in various clinical trials alone in NCT03119428 and NCT03628677, or in combination with anti-PD1 in NCT03119428 and NCT03628677, or with anti-PD-L1 in NCT03563716. Therefore, blocking the checkpoint receptor TIGIT represents a potential strategy to explore in immunotherapy.

### NKG2A

NKG2A is a cell surface inhibitory receptor expressed both on T cells and on NK cells, which forms a heterodimer with CD94 (108). In mice, about 40–60% of NK cells both in the peripheral and inside the tumors express NKG2A. In humans, more than half of NK cells are NKG2A^+^ not only in the blood, but also in tumors [e.g., squamous cell carcinoma of the head and neck (SCCHN)] (109). The expression of NKG2A on NK cells could be further up-regulated upon IL-15 stimulation (109). Importantly, among NKG2A^+^ NK cells, there is a subset co-expressing NKG2A and PD-1. On the other hand, non-classical MHC-I, HLA-E, is the ligand of NKG2A in humans, which is widely expressed by various types of tumors (e.g., lung, pancreas, stomach, colon, head and neck, and liver tumor tissues) (109). In mice, the ligand of NKG2A is Qa-1^b^. Binding of NKG2A/CD94 to its ligand suppresses the effector functions of T and NK cells by recruitment of the SHP-1 tyrosine phosphatase to the ITIM in the intracellular domain of NKG2A.

Blockade or abrogated expression of NKG2A rescued HLA-E-mediated suppression of cytotoxicity and IFN-γ production by NK cells *in vitro* (109, 110), and rendered NK cells with enhanced efficacy against HLA-E^+^ tumors *in vivo* upon infusion (110). Combined blockade of both NKG2A and PD-1 synergistically stimulated the degranulation of NKG2A^+^PD-1^+^ NK cells in HLA-E^+^PD-L1^+^ target cell co-culture (109). Disruption of NKG2A-Qa-1^b^ interaction by knocking out Qa-1^b^ on tumor cells promoted NK cell–dependent anti-tumor efficacy (109). Consistent with these functional studies, the expression of NKG2A and HLA-E in hepatocellular carcinoma (HCC) tissues correlated with poor prognosis of HCC patients (60).

Monalizumab, a humanized anti-NKG2A antibody, enhanced NK cell activity against various tumor cells and rescued CD8^+^ T cell function in combination with PD-1/PD-L1 axis blockade (109). Importantly, combined blockade of both NKG2A and PD-1/PD-L1 exhibited synergistic anti-tumor efficacy with improved survival compared with PD-1/PD-L1 blockade alone (109). This efficacy was shown to be NK-dependent, since NK cell depletion greatly shortened the prolonged survival of mice (109). In addition, since NKG2A is also up-regulated on CD8^+^ T cells in tumors, blockade of NKG2A would also stimulate CD8^+^ T cell–dependent responses (109).

Monalizumab also stimulated NK cell ADCC (antibody-dependent cell-mediated cytotoxicity) activity against antibody-coated target cells *in vitro* (109). In a phase II clinical trial NCT02643550 for the treatment of SCCHN, monalizumab combined with cetuximab, an anti-EGFR monoclonal antibody, resulted in a partial response rate of 31%, and stable disease at 54%, compared with the historical data of 13% objective response rate for single agent cetuximab, showing that NKG2A blockade has the potential of treating tumor patients in combination with tumor-targeting antibodies. Besides, NKG2A single blockade for the therapy of gynecologic malignancies (NCT02459301), or in combination with ibrutinib (a BTK inhibitor) for the therapy of chronic lymphocytic leukemia (CLL) (NCT02557516), are also under clinical trials.

### CD96

CD96 is a transmembrane glycoprotein Ig superfamily receptor expressed on T cells and NK cells. CD96 was earlier found to mediate the adhesion between NK cells and tumor cells to facilitate NK cell cytolysis (111). Later, the use of CD96^−/−^ mice revealed the role of CD96 as an important checkpoint for NK cell effector functions. Loss of CD96 rendered NK cells with hyper-production of IFN-γ in mice challenged with LPS (112). In chemical-induced tumor models, mice deficient in CD96 displayed more resistance to tumor growth in both an NK and IFN-γ –dependent manner (112). In HCC patients, the percentage and intensity of CD96 on NK cells, as well as the numbers of CD96^+^ NK cells, were higher for tumor-infiltrating NK cells compared with NK cells from peri-tumoral tissues (88). CD96^+^ NK cells are more severely dysfunctional compared with CD96^−^ NK cells, as evidenced by lowered expression of IFN-γ and TNF-α, as well as lower gene expression levels of *Tbx21, Prf1* and *Gzmb*, and increased gene expression levels of *Il-10* and *Tgf-*β (88). Importantly, high expression levels of CD96, or its ligand CD155 in tumors of HCC patients, was associated with poor disease prognosis (88).

Therapeutic blockade of CD96 in tumor metastasis models confirmed its role as a checkpoint receptor on NK cells. CD96 blockade was shown to inhibit experimental metastases in three different models (113). The efficacy was dependent on NK cells, CD226, and IFN-γ, but not dependent on activating Fc receptors (113). Furthermore, when combined with CD96 blockade, anti-CTLA-4, anti-PD-1, or chemotherapy was more effective. Co-blockade of CD96 and PD-1 resulted in increased local NK cell IFN-γ production and infiltration (113). These studies demonstrate that CD96 checkpoint blockade represents a potential immunotherapy strategy targeting NK cells.

### PD-1

Compared with the checkpoint receptors discussed above, the expression of PD-1 on NK cells is relatively minor. The levels of PD-1 on NK cells could be substantially up-regulated upon viral infections or in specific tissue/organs (114). In tumors in both humans and mice, NK cells displayed higher expression of PD-1 above baseline, although not at a high percentage (115, 116). PD-1^+^ NK cells, unlike TIGIT^+^, or CD96^+^ NK cells, displayed stronger effector potentials than PD-1^−^ NK cells, as shown by higher levels of IFN-γ and granzyme B upon IL-2 stimulation *in vitro* (116). However, in the tumor microenvironment where PD-L1 is expressed at high levels, this subset of PD-1^+^ NK cells might be dysfunctional under effects of the inhibitory signaling from interaction between PD-1 and PD-L1. Based on these studies, PD-1/PD-L1 blockade might therefore reverse the dysfunctional status of PD-1^+^ NK cells in this context, adding to the benefits from enhanced T cell responses upon PD-1/PD-L1 blockade.

### Cbl-b

Genetic deletion of the E3 ubiquitin ligase Cbl-b, or treatment with a small molecule targeting the substrate TAM tyrosine kinase receptors Tyro3, Axl and Mer, was shown to efficiently enhance anti-metastatic activity of NK cells in mouse models (47). In addition, the anticoagulant warfarin stimulated the anti-metastatic activity of NK cells via Cbl-b/TAM receptors (47). These data indicate that the Cbl-b/TAM pathway is a “checkpoint” that normally suppresses NK cell anti-tumor activity, and that therapeutically targeting this pathway might unleash the anti-metastatic potential of NK cells.

### IL-1R8

Interleukin-1 receptor 8 (IL-1R8), a negative regulator of Toll-Like and Interleukin-1 Receptor family signaling, is highly expressed on NK cells, and is increased during NK cells maturation (117). Also, IL-1R8 is expressed by tumor cells (e.g., in breast cancers) (118). Not only the expression of IL-1R8 in NK cells, but also that in the tumor cells, inhibited NK cell activation and NK -mediated control of tumor growth and metastasis, highlighting its role as a checkpoint for NK cell tumor immunity (117, 118). Therefore, therapeutically targeting IL-1R8 might represent a potential strategy to boost NK cell anti-tumor immunity.

### CIS

CIS is a negative regulator of IL-15 signaling by inhibiting the downstream JAK-STAT pathway. CIS expression was increased in NK cells upon cellular activation, such as in response to IL-15 (48). NK cells deficient in *Cish* showed increased JAK-STAT signaling, and enhanced proliferation, survival, IFN-γ production, and cytotoxicity against tumors (48). Mice deficient in *Cish* were resistant to melanoma, prostate, and breast cancer metastasis *in vivo* (48). The combination of *Cish* deficiency with targeted therapies or immune checkpoint blockade therapies displayed further improved control of metastasis (119). These data demonstrate that CIS acts as a potent intracellular checkpoint to target in NK cell-mediated tumor immunity.

### A2A Adenosine Receptor

Adenosine is an endogenous purine nucleoside that binds adenosine receptors. High levels of adenosine is present in the tumor microenvironment (120), impairing both the anti-tumor effector functions and maturation of NK cells (69, 121) via the A2A adenosine receptor on NK cells. Either inhibition of the adenosine -generating enzymes, CD73 or CD39, or blockade of A2A adenosine receptor, displayed immune stimulatory and anti-tumor effects in mouse models (122–124). Furthermore, a combination of A2A receptor inhibitors and PD-1 blockade significantly reduced metastasis of CD73^+^ tumors and prolonged the survival of mice compared with monotherapy alone (125, 126). Notably, the combination therapy depended on NK cells and IFN-γ, and to a less extent, CD8^+^ T cells (125). Recently, monoclonal antibodies targeting human membrane-associated and soluble forms of CD39 and CD73, respectively, were reported to efficiently block the hydrolysis of immunogenic ATP into immunosuppressive adenosine, and could restore the activation of cancer patient-derived T cells (127). Importantly, the CD39-inhibiting antibody increased the anti-tumor activity of the ATP-inducing chemotherapeutic drug oxaliplatin in a human CD39 knockin mouse preclinical model (127). Therefore, targeting the A2A adenosine receptor pathway can enhance NK cell anti-tumor activity, and might synergize with T cell–based checkpoint immunotherapy or immunogenic chemotherapy.

## Perspective

In conclusion, the well-documented role of NK cells in tumor surveillance has been further substantiated by recent progresses in NK-based checkpoint blockade immunotherapy, which targets NK cells to stimulate anti-tumor responses. More importantly, some strategies displayed the potentials to further improve current T cell–based immunotherapies. These studies indicate that NK-based immunotherapy represents a promising direction worthy of further investigations, especially in the current age of tumor immunotherapy.

Among these studies, while most have confirmed the roles of NK cells in controlling blood cancers and tumor metastasis, some have also proposed a role for NK cells in surveillance against solid tumors with evidence from either mouse models or clinical relevance, at least in some contexts (22). Based on this limited yet ever-growing evidence, it is not unreasonable to assume that NK cells might fully exhibit their anti-tumor effector potentials even in solid tumors, provided that we could be able to remove some of the either current or unknown checkpoints 1 day. In order to do so, apparently, a long way still lies ahead. Most of the studies on NK cell biology have been performed in normal mice, instead of in the immune suppressive landscape of tumors. As discussed in the above sections, intratumoral NK cells are mostly dysfunctional, and display alterations in many aspects compared with peripheral NK cells in normal mice. Not only NK-intrinsic biology, but also various NK-extrinsic factors from the tumor microenvironment governs the actual responses of intratumoral NK cells, making it difficult to interpret our basic knowledge for NK cell biology locally in the tumors. Therefore, more studies are required on the tumor regional features of NK cells.

In addition, the current age of immunotherapy urges the focus of investigations in tumor immunology to be on why so many patients are unresponsive to therapies, and how to increase the response rates. This requires us to look into the mechanisms regulating NK cell functions not only in the tumor immune suppressive landscape, but also in the settings of tumor immunotherapies, hopefully leading to novel strategies to improve current therapies.

Currently, the physiological roles in tumor surveillance by NK cells, as well as the therapeutic potential, of many surface receptors on NK cells have been demonstrated (e.g., KIR, TIGIT, NKG2A, CD96, and PD-1), while those of many others still remain to be shown (e.g., LAG-3 and TIM-3). For the ongoing interest in searching for novel “checkpoints” to target, it is important to describe the expression pattern of specific checkpoint receptors on NK cells (as well as their ligands on other cells), as well as on T cells, in specific tumors, in specific tumor stage, and in specific therapeutic settings. The underlying rationale is that only when the checkpoint (as well as its ligand) is expressed at functional levels, should the targeting be antagonizing its function. As we discussed in the above sections, checkpoint molecules display different expression patterns: (1) some are constitutively and stably expressed by NK cells; (2) some are normally absent or lowly expressed, and are up-regulated upon stimulation; (3) some are constitutively expressed normally, and are further up-regulated in special contexts. However, more detailed information is required. For example, in future studies on checkpoints, we also need to pay attention to whether the expression pattern of a specific checkpoint molecule is different in “hot” tumors and in “cold” tumors, and whether it is altered in hosts receiving anti-tumor therapies. The intensity of anti-tumor immune responses, as well as the immune suppression by the microenvironment, might be different under these different contexts, which might affect the expression levels of these checkpoint molecules. Therefore, detailed information is required for the complete description of the spatiotemporal expression profile of these checkpoint molecules. Only when we have sufficient knowledge about the immune checkpoint landscapes of specific tumor microenvironments could we rationally design therapeutic strategies precisely targeting the functional checkpoints for the proper indications at the right time.

Collectively, accumulating evidence has indicated a role of NK cells in surveillance against not only blood cancers and metastasis, but also solid tumors, at least in some contexts. In recent years, a lot of progress has been made regarding the role of NK cells, as well as the role of NK cell checkpoints in anti-tumor immunity. Targeting those checkpoints displayed the potential of boosting NK cell activity against tumors. Importantly, some might improve current T cell–based checkpoint immunotherapies. Although a lot remains to be understood, recent studies demonstrate the promise that further investigations into the regional features of NK cells in tumors might give rise to novel checkpoint immunotherapies in future.

## Author Contributions

JB and ZT conceived and wrote the manuscript.

### Conflict of Interest Statement

The authors declare that the research was conducted in the absence of any commercial or financial relationships that could be construed as a potential conflict of interest.
